# Decratonization by rifting enables orogenic reworking and transcurrent dispersal of old terranes in NE Brazil

**DOI:** 10.1038/s41598-021-84703-x

**Published:** 2021-03-11

**Authors:** Carlos E. Ganade, Roberto F. Weinberg, Fabricio A. Caxito, Leonardo B. L. Lopes, Lucas R. Tesser, Iago S. Costa

**Affiliations:** 1 Geological Survey of Brazil/CEDES, Rio de Janeiro, Brazil; 2 School of Earth, Atmosphere and Environment, Monash University, Clayton, Australia; 3 Universidade Federal de Minas Gerais, Belo Horizonte, Brazil; 4 Universidade de São Paulo, São Paulo, Brazil

**Keywords:** Geochemistry, Geodynamics, Tectonics

## Abstract

Dispersion and deformation of cratonic fragments within orogens require weakening of the craton margins in a process of decratonization. The orogenic Borborema Province, in NE Brazil, is one of several Brasiliano/Pan-African late Neoproterozoic orogens that led to the amalgamation of Gondwana. A common feature of these orogens is that a period of extension and opening of narrow oceans preceded inversion and collision. For the case of the Borborema Province, the São Francisco Craton was pulled away from its other half, the Benino-Nigerian Shield, during an intermittent extension event between 1.0–0.92 and 0.9–0.82 Ga. This was followed by inversion of an embryonic and confined oceanic basin at *ca.* 0.60 Ga and transpressional orogeny from *ca.* 0.59 Ga onwards. Here we investigate the boundary region between the north São Francisco Craton and the Borborema Province and demonstrate how cratonic blocks became physically involved in the orogeny. We combine these results with a wide compilation of U–Pb and Nd-isotopic model ages to show that the Borborema Province consists of up to 65% of strongly sheared ancient rocks affiliated with the São Francisco/Benino-Nigerian Craton, separated by major transcurrent shear zones, with only ≈ 15% addition of juvenile material during the Neoproterozoic orogeny. This evolution is repeated across a number of Brasiliano/Pan-African orogens, with significant local variations, and indicate that extension weakened cratonic regions in a process of decratonization that prepared them for involvement in the orogenies, that led to the amalgamation of Gondwana.

## Introduction

Cratons are old, stable continental regions with thick buoyant keels that resist deformation. Their thick and cold refractory lithospheric keel is taken to be responsible for their integrity, protecting them from tectonic reworking^1^. However, during continental collisions, colliding cratonic blocks can be partially reworked to generate continental tracts that are no longer cratons but that are not typical orogens either^2^. Thus, in order to understand the origin of these old continental crustal tracts within orogenies, it is necessary to address the specific mechanisms for both the weakening of the craton lithosphere (*c.f.* decratonization) and their subsequent reworking. First order controls in the reworking of these strong lithospheres are temporal and spatial variations of their thermal state and pre-existing mechanical anisotropies or major compositional boundaries^3–8^. The North China Craton is a classic example of decratonization where lithospheric thinning, asthenospheric decompression and magmatism changed the thermal and chemical state of the cratonic lithosphere resulting in wholesale decratonization^9–12^.

**Figure 1:**
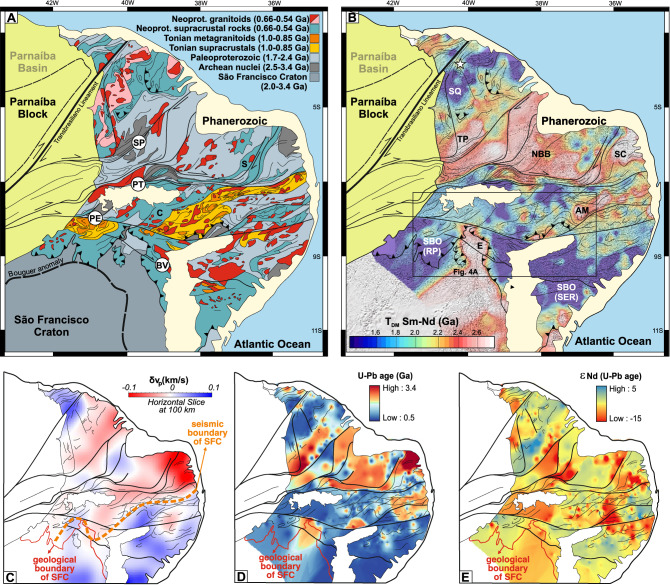
Age of lithological units and distribution of model ages in the Borborema Province. (**A**) Interpreted geological-age map of the Borborema Province. Labels correspond to names of shear zones. Geological base maps are from the Geological Survey of Brazil and freely available at http://geosgb.cprm.gov.br. (**B**) Sm–Nd T_DM_ age map of the Borborema Province and northern São Francisco Craton with magnetic first derivative image as background (see Fig. S1 for data point location). Note the Entremontes promontory (labelled E), a part of the SFC separating two fold-and-thrust belts of the Southern Borborema Orogen. *SP* Senador Pompeo shear zone, *PT* Patos shear zone, *PE* Pernambuco shear zone, *BV* Boa Vista shear zone, *SQ* Santa Quitéria arc, *TP* Tróia-Pedra Branca terrane, *NBB* Northern Borborema block, *SC* São José do Campestre terrane, *AM* Alto Moxotó terrane, *E* Entremontes block, *SBO* Southern Borborema Orogen, *RP* Riacho do Pontal fold-and-thrust belt, *SER* Sergipano fold-and-thrust belt, *C* Cachoeirinha Belt, *S* Seridó Belt. (**C**) P-wave tomography model for the BP at 100 km depth^35^ showing a markedly high velocity under the Alto Moxotó terrane. (**D**) Spatial distribution of zircon U–Pb crystallization ages of igneous and metaigneous rocks of the Borborema Province. (**E**) Spatial distribution of ε_Nd__(t)_ values calculated for the time of crystallization shown in (**D**). The Sm–Nd T_DM_ ages, U–Pb ages and ε_Nd(t)_ values were gridded in the ArcGIS software using Inverse-Distance-Weighted Interpolation (IDW).

Here we describe the process of decratonization of the São Francisco Craton (SFC) during the Neoproterozoic in northeast Brazil. This craton is bound to the north by the late Neoproterozoic orogenic Borborema Province (BP). In the province, pre-orogenic extension started as early as 1.0 Ga and resulted in the development of intracontinental extensional basins^13^, passive margin basins flanking the craton^14–16^, and separation of the SFC from the African Benino-Nigerian Shield^17,18^. Remarkably, this orogenic province includes a number of reworked and deformed Archean-Paleoproterozoic terranes among the Neoproterozoic sedimentary basins and granitic intrusions^19^. These old terranes are relatively small (10,000 to 40,000 km^2^), bounded by large-scale continental shear zones^19,20^ (Fig. 1A and B), and their affinity with the SFC have been previously proposed^21^. Although no quantitative crustal growth curves of the orogenic Borborema Province have been reported, present models range from dominantly intracontinental reworking^21,22^, to arc accretion^23,24,37^ and docking followed by reworking of unrelated terranes^25^, or even to questionable autochthonous accretion surrounding old small reworked Archean crust^26^.

The orogenic Borborema Province (BP) comprises two separate and interacting collisions: one on the west side, part of the vast West Gondwana Orogen^27^, and one in the south, against the SFC, and termed here the Southern Borborema Orogen. These two collisions were partly contemporaneous and interacted involving the entire BP. In this paper we will use the term BP to refer to the whole orogenic area. The current tectonic configuration of the province is controlled by these collisions^19^. Their tectonic nature is indicated by the record of UHP metamorphism and eclogites^27,28^, marginal ophiolites^29,30^, and arc sequences^16,24,﻿37﻿^, that together suggest the West Gondwana Orogen resulted from oceanic subduction followed by collision between the Benino-Nigerian Shield and the West African Craton^19,27^, while the Southern Borborema Orogen resulted from collision of the BP against the SFC. The two different orogens interacted in a complex collisional zone, giving rise to an intricate network of continental shear zones that controlled deformation^19^. In this context, the large Transbrasiliano-Kandi shear zone, that developed as a result of the collision along the West Gondwana Orogen, acted as a dextral transfer zone leading to BP collision against the SFC and the development of the Southern Borborema Orogen^17^.

In this paper, we compile large volumes of whole-rock Nd isotope data, zircon U–Pb geochronology data, as well as geological and geophysical data from the BP and northern part of the SFC and the Benino-Nigerian Shield. We use the data to first quantify the Neoproterozoic continental growth of the BP and establish the dominance of recycled cratonic material, and then to investigate how the decratonization allowed former cratonic Archean-Paleoproterozoic terranes to be entrained in the BP during the Neoproterozoic Orogeny.

## Geological setting of the Borborema Province

The distribution of rocks in the triangular wedge-shaped orogenic BP (Fig. 1) was ultimately controlled by a set of Neoproterozoic strike-slip shear zones^19,20^. These shear zones bound the north, central and south sub-provinces and within them several other informal domains^32^. Shearing was accompanied by granitoids with intrusions concentrated at 0.59–0.56 Ga^19,22^. Archean and Paleoproterozoic rocks are found as basement inliers, often referred as blocks and/or terranes, all over the BP, and always bounded by Neoproterozoic shear zones (Fig. 1). Early Neoproterozoic extensional events starting as early as 1.0 Ga are marked by granitoid intrusions, bimodal volcanism and deposition of immature terrigenous and minor carbonatic sediments during the so-called Cariris Velhos event^13^. This event was initially defined as orogenic^33^, however no associated Tonian deformation and metamorphism have been reported, which led to the proposition that this was an extensional event^21^. In the southern BP, this event culminated in the development of a passive margin sequence along the edge of the SFC associated with 0.9–0.82 Ga mafic–ultramafic intrusions, proximal margin-type ophiolites and A-type granites^14,16^. Early Neoproterozoic passive margin and intracontinental sedimentation was overlain by orogenic sedimentary successions as young as 0.58 Ga^16^ contemporaneous with other basins within the central and north BP^34^. The lithological boundary of the SFC and BP is defined in many geological maps (Fig. 1A), however seismic data indicate that the lithospheric cratonic signature at 100 km depth extends further north ending within the central portion of BP^35,36^ (Fig. 1C).

During the Late Neoproterozoic, from 0.65 to 0.61 Ga, predating continental collisional events, continental magmatic arcs developed in the west^24^ and south^37^ parts of the province, closing intervening oceanic basins. During the collisions that ensued, the province was squeezed between two impinging continents, one continent coming in from the west and the other, the São Francisco Craton, coming in from the south^19^. As a consequence, the BP tectonically “escaped” obliquely with the development of a throughgoing network of transcurrent shear zones^19,20^ that both shear and bound Archean-Paleoproterozoic terranes^38–40^, such as the Alto Moxotó terrane^25^ (Fig. 1). Just how these old terranes became involved in the orogen remains unclear.

## Results

### U–Pb ages, Nd isotopic spatial distribution and crustal growth

**Figure 2:**
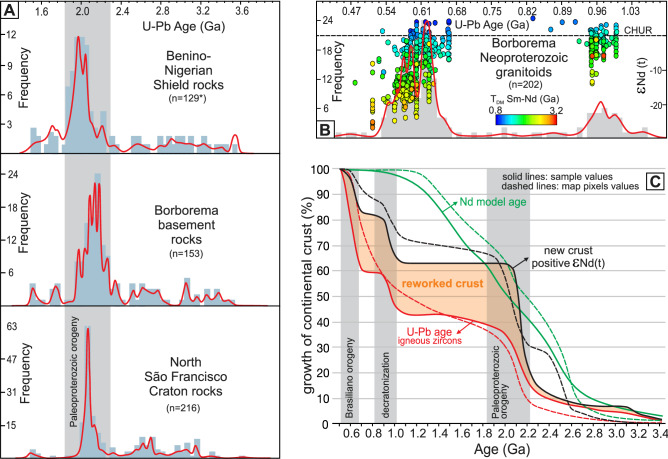
Zircon U–Pb, Nd whole-rock data and crustal growth. (**A**) Similarity of zircon U–Pb crystallization age distribution for the North São Francisco Craton, Benino-Nigerian Shield and Borborema Province (see “Methods” section for explanations on data compilation). (**B**) Zircon U–Pb crystallization ages for the Neoproterozoic magmatism in the Borborema Province and their ε_Nd(t)_ values and T_DM_ (given by the colour of the circles). Data show increased involvement of old crust during the Brasiliano Orogen, as oceanic basins closed, and collision progressed (see “Methods” section for data compilation). (**C**) Continental growth for the Borborema Province using data from samples of igneous and metaigneous rocks (solid lines) and exposed area coverd by these rocks based on pixel values from the maps in Fig. 1D, E (dashed lines). Colour of the lines represent cumulative U–Pb ages (red), ε_Nd(t)_ < 0 (black) and Nd model ages (green), binned for each 100 Myr interval from 3.4 to 0.5 Ga. Growth is estimated from the cumulative proportion of juvenile crustal addition using the ε_Nd(t)_ cut-off value of zero for the igneous and metaigneous rock samples and gridded pixels for the crystalline igneous and metaigneous areas from the maps in Fig. 1D, E (see “Methods” section).

The U–Pb ages of igneous rocks in the northern SFC and Benino-Nigerian Shield show a similar pattern to those of the ancient basement terranes in the BP, recording similar episodes of magma production during the Archean and most intensely during the Paleoproterozoic (Fig. 2A, Supplementary Data 1). In the Neoproterozoic, U–Pb ages of igneous rocks from the BP show two intervals of magma production at 1.0–0.92 Ga and 0.67–0.52 Ga, with a few ages < 0.50 Ga (Fig. 2B). Both intervals are characterized by production of new juvenile crust combined with recycling of old pre-existing crust. The spatial distribution of the interpolated U–Pb ages and ε_Nd(t)_ values (Fig. 1D and E), excluding the areas covered by sedimentary and metasedimentary rocks, was used to estimate the crustal growth during the evolution of the Borborema Province (see “Methods” section, Supplementary Fig. S1).

The gridded values resulted in a map with 46,426 pixels with a pixel size of 3.2 × 3.2 km (pixel area = 10.24 km^2^). These pixels where clipped to the crystalline basement area of the Borborema Province, excluding the Neoproterozoic metasedimentary belts, Phanerozoic basins and more recent cover, resulting in a map with 17,235 pixels with a total area of 176,486 km^2^. Pixel values of Nd model ages (Fig. 1B) were binned into 100 Myr interval, from 0.5 to 3.4 Ga, and so were the U–Pb crystallization ages from individual rock samples. Positive ε_Nd(t)_ values were used to distinguish juvenile addition of new crust to the Borborema Province through time.

The results based on the exposed surface area, incorporating both zircon U–Pb and Nd isotopes, indicate that 65–60% of the province was already formed by the end of the Paleoproterozoic, with a rapid growth rate between 2.3 to 2.0 Ga, when 55–50% of the continental crust of the Borborema Province formed (Fig. 2C). During the Early Neoproterozoic, from 1.0 to 0.7 Ga, new juvenile additions accounts for ~ 20% of the crust. This period includes the early stages of the Santa Quitéria arc at *ca.* 0.85 Ga^24^ and magmatism related to the Cariris Velhos event from 1.0 to 0.92 Ga^41^. The production of new continental crust during the main Neoproterozoic orogenic period, between 0.67 and 0.52 Ga, accounts for only ~ 15% of the new continental crust in the province, suggesting the orogeny was characterized by whole-sale lithospheric reworking with minor juvenile magma input.

**Figure 3:**
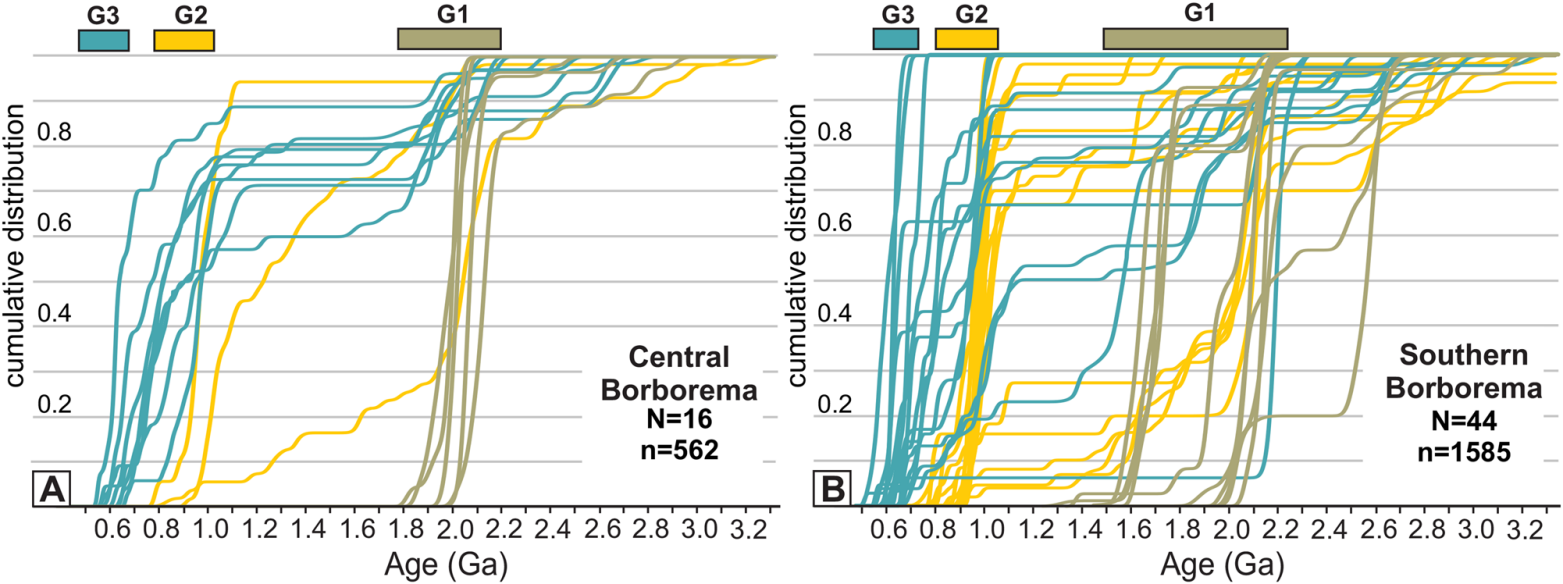
Provenance patterns of sedimentary rocks from central and south Borborema Province. (**A**) Cumulative probability plot showing the distribution of U–Pb ages from detrital zircon grains for samples from the central Borborema Province (region between the Pernambuco and Patos shear zones in Fig. 1). (**B**) Same as A, but for the southern Borborema Province (region between Pernambuco and the northern margin of the São Francisco Craton in Fig. 1). The distributions show three different patterns denoting different source areas: G1 cratonic provenance only, G2 Tonian extensional magmatic rocks plus cratonic provenance, and G3 Neoproterozoic orogenic magmatic rocks plus a variety of older sources. N is the number of rock samples; n is the total number of zircon grains analyzed. The bars on top of the diagrams depict the range of the youngest zircon grains found in each group.

The compiled dataset of detrital zircons, including 2147 grains from 60 samples (Supplementary Data 2) from the central and southern Borborema Province, defines three main groups (G1, G2 and G3) based on the youngest zircon age and source areas given by the older zircon dates in each group (Fig. 3A,B). The first group (G1) is dominated by samples with youngest zircon ages > 1.4 Ga and source areas with dominantly Paleoproterozoic and Archean ages. The second group (G2) is characterized by samples with youngest zircon grains between 0.7 and 1.0 Ga with source areas dominated by rocks ranging from 0.9 to 1.0 Ga. A subgroup of samples in G2 also shows source areas dominated by Paleoproterozoic and Archean rocks such as those in G1. The last group (G3) comprises samples with youngest zircon ages between 0.5 and 0.7 Ga, representing the youngest sedimentary record in the province, associated with syn-orogenic sedimentation. The source areas of G3 vary from rocks with ages in the range of 0.65 to 0.52 Ga, such as the foreland strata of the Sergipano fold-and-thrust belt^16^, to rocks with source components from 1.0 to 0.65 Ga and even older (Fig. 3A and B).

### Transpression and correlation of terranes

**Figure 4:**
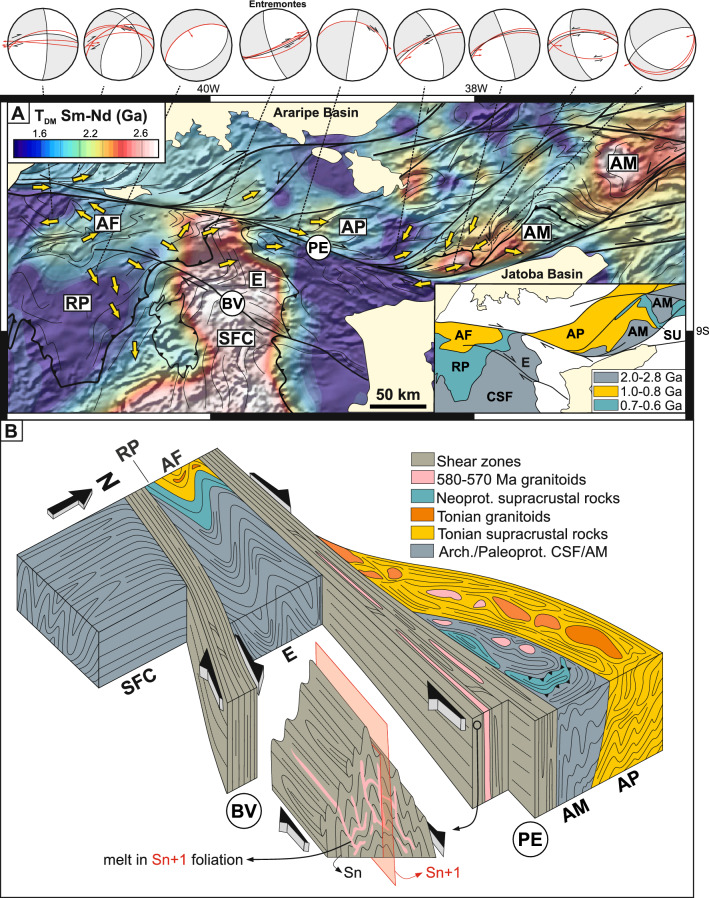
Transpression and correlation of terranes in the central and southern Borborema Province. (**A**) Sm–Nd T_DM_ age map of the south Borborema Province and northern edge of the São Francisco Craton with magnetic first derivative image as background. Inset illustrates dextral displacements of the 1.0 to 0.8 Ga and the older 2.8–2.0 Ga terranes across the Pernambuco shear zone, resulting from transcurrent tectonics. The yellow arrows indicate the direction of tectonic transport. *BV* Boa Vista shear zone, *PE* Pernambuco shear zone, *E* Entremontes block, *AF* Afeição domain; *RP* Riacho do Pontal fold-and-thrust belt; *AP* Alto Pajeú terrane; *AM* Alto Moxotó terrane, *SFC* São Francisco Craton. The stereograms report the Sn + 1 foliation along with its stretching lineation (red arrows) and shear sense when available. The Sm–Nd T_DM_ ages were gridded in the ArcGIS software using Inverse-Distance-Weighted Interpolation (IDW). (**B**) Block diagram illustrating the transpressive regime in the Entremontes block with progressive displacement and increased deformation from the craton to the Entremontes block and to the Alto Moxotó terrane across the Boa Vista (*BV*) and the Pernambuco (*PE*) shear zones. Note how the Tonian rocks follow the same increase in deformation from the Afeição (*AF*) domain to the Alto Pajeú (*AP*) terrane. Together, they indicate a dextral displacement across the Pernambuco shear zone of ≈ 200 km.

Another aspect revealed by our data compilation and geophysical image investigation is the link between old Paleoproterozoic and Archean terranes across the province. The Southern Borborema Orogen (Figs. 1A,B, 4), is split into the Sergipano and Riacho do Pontal fold-and-thrust belts by a promontory of the São Francisco Craton, well-defined in the Nd isotopic model age map (Fig. 1B) and geological maps^15,16^. This promontory forms the Entremontes block and may mark an inherited feature of the paleocontinental passive margin inverted during onset of the Southern Borborema Orogen. It comprises mainly Archean and Paleoproterozoic gneisses, migmatites and supracrustal rocks^42^.

The combination of our field data, geological maps, isotopic data and geophysical images show that the Entremontes block is bound by the Pernambuco shear zone in the north and the Boa Vista shear zone in the south (Fig. 4A). Movement on these shear zones forced rotation and internal deformation of the block under a transpressive regime (Fig. 4B) leading to the folds documented in the aeromagnetic images at wavelengths of 5 to 20 km with 2D axial planes subparallel to the shear zones (Fig. 4B). In outcrop, folds are tight and fold a previous foliation (Sn), generating a steep axial plane (Sn + 1) permeated by leucosomes indicative of syn-tectonic partial melting (Fig. 4B). Stretching lineation has low rake and is commonly parallel to the fold axes, plunging at low angles to WSW or ENE. Kinematic indicators on Sn + 1 planes indicate consistent top-to-NE or E transport in the Entremontes block (yellow arrows in Fig. 4A). This direction could either be a result of rotation of early-formed shear zones, or formed contemporaneously with transcurrent movement, defining a constrictional transpressional belt. The metasedimentary Riacho do Pontal fold-and-thrust belt, west of the Entremontes block, includes the Afeição domain comprising Tonian metasedimentary and metaigneous rocks. In contrast to the Entremontes, the direction of tectonic transport for the entire Riacho do Pontal belt is dominantly top-to SW, thrusting the belt over the SFC^43^ (Fig. 4A).

The geophysical and Nd isotopic characteristics of the ancient Entremontes block and the Tonian-age Afeição domain can be recognized in the central Borborema Province, north of the Pernambuco shear zone, displaced dextrally by ≈ 200 km and deformed into sigmoidal terranes characteristic of the BP (Fig. 4, Supplementary Fig. S1). U–Pb ages and ε_Nd(t)_, as well as K-Th-U contents, as recorded in the radiometric images (Supplementary Fig. S1), show that the Afeição domain^43^ can be linked with the contemporaneous Alto Pajeú terrane^13^, and the Entremontes block can be linked with the Alto Moxotó terrane, also composed of Paleoproterozoic and Archean gneisses and migmatites^25,42^. The Alto Moxotó terrane includes high-grade Late Neoproterozoic metasedimentary rocks of the Surubim Complex that can be correlated to the lower-grade equivalents of the Riacho do Pontal fold-and-thrust belt and of the Cabrobó Complex overthrusting the Entremontes block. The Surubim Complex and these lower grade-rocks share similar detrital zircon age signatures^22,42,43^.

In summary, much of the Borborema Province has old Nd model ages and zircon population signatures similar to the São Francisco Craton/Benino-Nigerian Shield (Figs. 1 and 2A), suggesting direct or indirect derivation from these areas. The stepwise process of breakdown and involvement of cratonic blocks is preserved at the margin of the craton, where increased deformation of cratonic blocks is recorded across the Pernambuco shear zone and associated with a dextral displacement of ≈ 200 km.

## Discussion: decratonization, terrane dispersion and reworking

**Figure 5:**
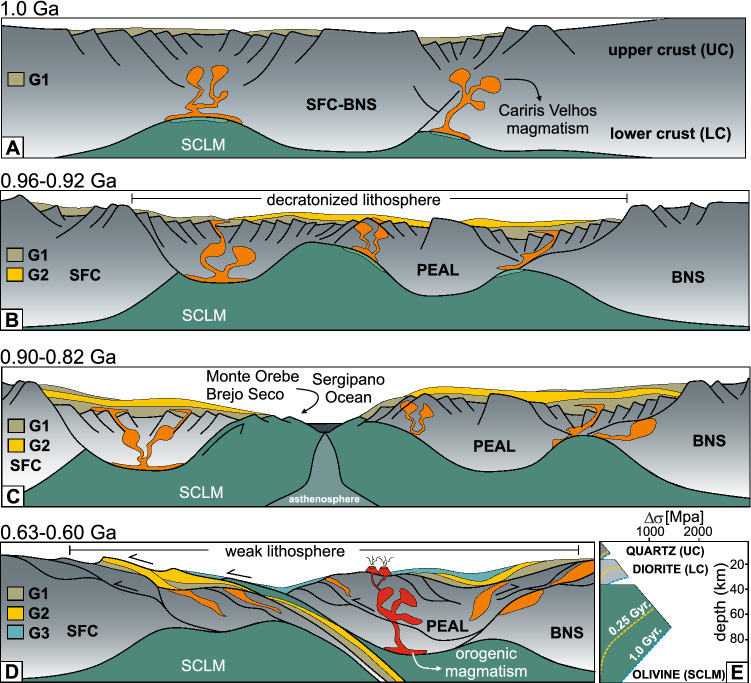
Tectonic evolution and formation of the northern São Francisco passive margin. (**A**) Initial extension and partial melting of the sub-continental lithospheric mantle (SCLM) generating mafic magmas associated with the early Cariris Velhos granitoids and filling of depocenters with sediments shed by cratonic sources (G1 pattern of detrital zircon ages in Fig. 3). (**B**) Continuous extension and necking of the cratonic lithosphere associated with intrusion of late Cariris Velhos granitoids, extrusion of volcanic rocks and sedimentation of strata derived from erosion of Cariris Velhos magmatic rocks as well as cratonic rocks (G2 pattern of detrital zircon ages in Fig. 3). (**C**) Breakup of the cratonic lithosphere and formation of the PEAL continental ribbon and separation of the Benino-Nigerian Shield (*BNS*). This stage marks development of the São Francisco Craton passive margin accompanied by marginal mafic–ultramafic rocks of the Brejo Seco^14^ and Monte Orebe^29^ units interpreted as exhumed sub-continental lithospheric mantle. At this stage the margin is supplied by mixture of detrital material from the craton and Cariris Velhos magmatism. (**D**) Inversion of the passive margin basin and initial collision of the São Francisco Craton against the PEAL. At this stage sedimentary strata are dominated by Brasiliano-age orogenic detritus as well as detritus from the older sequences (G3 pattern of detrital zircon ages in Fig. 3). (**E**) Yield stress envelope^54^ for continental lithosphere as a function of the time since the last thermotectonic event (1.0 Ga: dashed blue lines) and (0.25 Ga: dashed yellow lines, corresponding approximately to the time gap between Tonian extension and start of the orogeny in the BP). The stress envelopes show that during the Brasiliano orogenic event, the cratonic area would be stronger than the regions that underwent the more recent Tonian thermotectonic event. This implies that the orogenic deformation would focus on the weaker decratonized lithosphere.

In the Borborema Province, crustal extension is marked by mafic–ultramafic intrusions (*e.g.,* the *ca.* 0.90 Ga Brejo Seco Unit^14^ and the *ca.* 0.82 Ga Monte Orebe Unit^29^), continental rift-like basic volcanic rocks (*e.g.,* the *ca.* 0.88 Ga Paulistana Complex^15^) and A-type orthogneisses (e.g., the *ca.* 0.87 Ga Pinhões pluton^44^), following intraplate 1.0–0.92 Ga A-type granitoids and bimodal volcanism^21,41^. Together this magmatism defines the Cariris Velhos event that records the initial extension of cratonic lithosphere^21^ (Fig. 5A,B). Mafic dikes of the Bahia-Gangila LIP intruding the north São Francisco Craton at 0.92–0.90 Ga^45^ further support the interpretation that this was a widespread extensional event. Depocenters in this dynamic extensional environment were favorably filled with detritus shed by the topographic highs of the cratonic lithosphere and/or by local supply of the 1.0–0.87 Ga igneous rocks, giving rise to the detrital zircon pattern observed in groups G1 and G2 (Figs. 3 and 5B). However, sampling of older Paleoproterozoic metasedimentary rocks can also generate the G1 pattern. The geochemical signature^14,29^ of the Brejo Seco and Monte Orebe units, tectonically emplaced in the distal portion of the SFC passive margin, points to a continental rather than an oceanic origin, typical of continental margin ophiolites of Ligurian and Western Alpine examples^46^. The last pulses of rifting are recorded by the 0.72–0.68 Ga ﻿mafic–ultramafic intrusive rocks of the Canindé Group in the Southern Borborema Province^16^, but the relation to the development of the SFC passive margin is still contentious. The absence of suprasubduction-type ophiolites (*e.g.,* Troodos ophiolite in Cyprus^46^), suggest that extension did not lead to the formation of a large ocean separating the rifted blocks. Instead, the break-up of the greater São Francisco Craton/Benino-Nigerian Shield resulted in local sub-continental lithospheric mantle (SCLM) exhumation followed by formation of an embryonic narrow ocean, the Sergipano Ocean^16^ (Fig. 5B and C). This is similar to the Alpine-Apennine poorly evolved oceanic basins, as also described in other Neoproterozoic passive margins^47^. If this is the case, the extensional event between 1.0 and 0.82 Ga must have been intermittent so as to prevent the opening of a large ocean. This intermittent extension pulled away crustal ribbons from the cratonic margin, such as the Pernambuco-Alagoas terrane (PEAL, Fig. 5C), that were subsequently deformed during inversion and final collision (Fig. 5D).

The break-up of the conjoined São Francisco Craton/Benino-Nigerian Shield during extension indicates that decratonization started already in the Early Neoproterozoic, in a manner akin to the Mesozoic decratonization of the North China Craton^9^, where extension associated with metasomatism^10^ reactivated and replaced lithospheric mantle by asthenosphere^48^ and facilitated continental breakup^49^. This hypothesis is supported by alkaline volcanism (*e.g.*, kimberlites) in the northern SFC from 1.15 to 0.68 Ga^50–52^.

The long-term effect of extension on lithospheric strength depends on the relative thinning of the crust in relation to the sub-continental lithospheric mantle (SCLM)^53,54^. Starting from a thick Archean lithosphere, as for the SFC^55,56^, Tonian thinning and refertilization of the SCLM (indicated by the alkaline volcanism), would have led to weakening that persisted long-after extension ended. Only after 0.40 to 0.75 Gyr from a given thermotectonic event, the temperature distribution in the continental lithosphere approaches equilibrium and stops evolving with time^53^ (Fig. 5E). Thus, the Tonian extensional event would have made sections of the cratonic lithosphere amenable to subsequent reworking within the orogenic realm.

**Figure 6:**
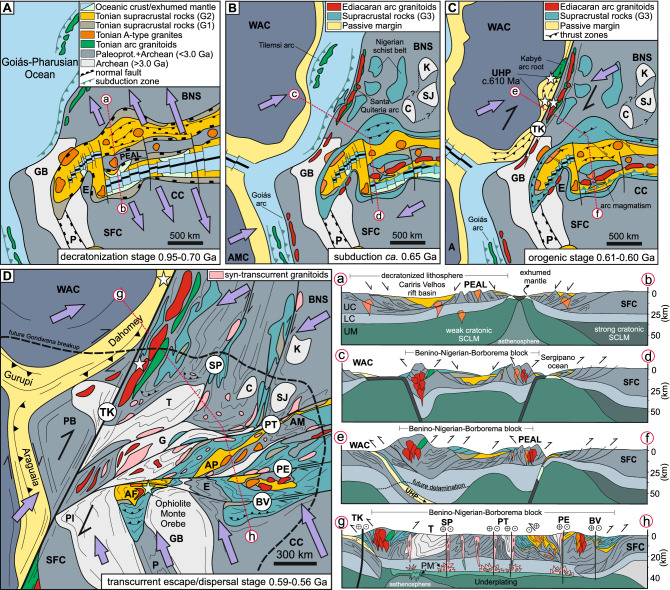
Neoproterozoic decratonization, transcurrent tectonics and terrane dispersion in NE Brazil. (**A**) Overall extension of the São Francisco Craton/Benino-Nigerian Shield (*SFC* and *BNS*), following intrusion of intraplate Cariris Velhos granitoids (1.0–0.92 Ga in orange) and leading to development of Tonian rift basins in restricted oceanic realms at 0.85–0.70 Ga (*c.f.* Sergipano Ocean recorded by the *ca.* 0.82 Ga Monte Orebe ophiolite^28^ (blue star in C) and aulacogens such as the 0.9–0.5 Ga Paramirim (*P*). Inception of peripherical intraoceanic and transitional arcs in the west along the Goiás-Pharusian Ocean. The Gavião Block (*GB*) is the old Archean cratonic core of the conjoined São Francisco Craton (*SFC*), Congo Craton (*CC*) and the Benino-Nigerian Shield (*BNS*). The Pernambuco-Alagoas terrane (*PEAL*) represents a continental ribbon of SFC rocks within the rift, that was extensively intruded by Neoproterozoic granitoids. Note the Entremontes (*E*) promontory on the paleomargin of the SFC. (**B**) Subduction stage marked by intrusion of continental arc related granitoids such as the Santa Quitéria and Major Isidoro/Betania arcs in the western and southern Borborema Province, respectively. Also shown are the Archean domains of Kaduna (*K*), Campo Grande (*C*) and São José do Campestre (*SJ*) and the Nigerian schist belts of the Benino-Nigerian Shield. (**C**) Overall shortening of the interior basins (Tonian rift + Brasiliano/Pan-African Ediacaran orogenic-related basins) as a response to the oblique continental collision along the West Gondwana Orogen (*WGO*) marked by ultra-high-pressure (UHP) metamorphism, and nucleation of the dextral Transbrasiliano-Kandi shear zone (*TK*)^17,19^ as a result of continental collision in the west. This stage is also marked by continental arc magmatism (0.63-0.60 Ga) in the Southern Borborema Orogen^16^ and shearing along the inactive Santa Quitéria arc^17^. (**D**) Zoom on NE Brazil and NW Africa illustrating final shortening, inversion of rift basins and development of the Borborema transcurrent shear zone system as a consequence of the double cratonic indentation associated with continued convergence of the West African Craton against the greater São Francisco-Saharan paleocontinent in the west and a counterclockwise rotation of the São Francisco Craton in the south. Transpression deformed and dispersed terranes sourced from the SFC within the Borborema Province. Transcurrent shear zones are associated with crustal heating and magmatism associated with delamination of the lithospheric mantle and asthenospheric upwelling^80,81^ that facilitated lateral crustal flow. Position of the cross-sections (on the right-hand-side of D) are indicated by pink dashed lines in A to D. Arrows and their size indicate direction and relative magnitude of the stresses, respectively. Shear zones: *TK* Transbrasiliano-Kandi, *PE* Pernambuco, *PT* Patos, *SP* Senador Pompeu, *BV* Boa Vista. Disrupted Tonian and cratonic domains: *AF* Afeição, *AP* Alto Pajeú, *AM* Alto Moxotó, *T* Tróia, *G* Granjeiro, *PI* Cristalândia do Piauí. *PM* in cross section (g) to (h) is partial melting.

This weakened, decratonized lithosphere between the Benino-Nigerian Shield and the São Francisco Craton (Fig. 6A) was reworked in two steps during the construction of the Borborema Province Orogen. The first step was a result of oblique continental collision with the West African Craton being thrust under the Benino-Nigerian Shield^17,27,57,58^. This was preceded by oceanic lithosphere subduction and magmatism in the aforementioned Santa Quitéria arc, and terminal collision led to the development of the dextral Transbrasiliano-Kandi strike-slip belt (Fig. 6B and C). The second step was a result of dextral movement on the Transbrasiliano-Kandi strike-slip belt. This movement brought the Benino-Nigerian Shield closer to the São Francisco Craton deforming the weakened decratonized area in between these two stiffer lithospheric domains and leading to the transpressional Borborema Province Orogeny (see cross-sections in Fig. 6D). The closure of the small intervening Sergipano Ocean (Fig. 5C), prior to collision and transpression, resulted in voluminous magmatism between 0.64 and 0.60 Ga, especially in the PEAL terrane and Sergipano fold-and-thrust belt^16,37^. This abundant magmatism could have resulted from increased H_2_O flux released from the subduction of sediments and serpentinized mantle from the extended margin of the approaching SFC, in a similar manner to that described in the transition from subduction to continental collision in the West Gondwana Orogen^59^. Erosion of this orogenic system contributed to the 0.65-0.52 Ga sedimentary input in the Southern Borborema Orogen, as observed in detrital zircon pattern of group G3 (Figs. 4 and 5B), while orogenic contribution in the central Borborema Province could represent a mixing between the Southern Borborema and West Gondwana Orogens.

The major transcurrent shear zones that characterize the Borborema Province splay out of the Transbrasiliano-Kandi shear zone^19^ and deformed the weakened cratonic margin. Deformation was a result of these two quasi-contemporaneous collisions^19,27^ (Fig. 6D). In the north part (north of the Patos shear zone in Fig. 1B), old Paleoproterozoic and minor volumes of Archean rocks dominate and represent the reworked southern continuation of the Benino-Nigerian Shield^17^. To the south, the Borborema Province records the interaction of the decratonized lithosphere and the São Francisco Craton. In this region, the transcurrent shear zones wrenched the blocks of the northern margin of the São Francisco Craton previously weakened by the Cariris Velhos extension. Deformation included a number of continental ribbons (*e.g.,* PEAL terrane) pulled away from the craton, and the intervening Tonian sedimentary basins (Fig. 6C and D).

The exact geometry of the decratonized terrane at the start of wrenching, and the source region of individual blocks now embedded in the BP remain undetermined. However, using rock ages, isotopic and geophysical signatures, we have linked the Tonian-age Afeição domain and the ancient Entremontes block south of the Pernambuco shear zone, with the Tonian-age Alto Pajeú and the ancient Alto Moxotó terranes, north of the shear zone. This implies ≈ 200 km dextral wrenching across the Pernambuco shear zone (Fig. 4). The rocks south of the shear zone record only incipient deformation, whereas those to the north are intensely strained into sigmoidal terranes, in harmony with the regional transcurrent deformation and the geological-geophysical structure of this region.

It is also possible to recognize signatures of specific sections of the SFC in the old blocks now within the BP. For example, the geological features of the Gavião Block in the SFC and of the Kaduna Massif in the Benino-Nigerian Shield^62^ can be found in blocks within the Central and Northern Borborema Province^38,40,60^. These features include rare occurrences of Paleoarchean rocks (> 3.4 Ga) embedded in Neoarchean (2.8–2.6 Ga) to Paleoproterozoic (2.1–2.0 Ga) rocks imprinted by *ca.* 2.0 Ga high-grade metamorphism^60,61^. We conclude that the stepwise increase in deformation intensity of craton margin blocks, illustrated by the Entremontes block and the sigmoidal-shaped Alto Moxotó terrane, illustrates how a number of other Archean-Paleoproterozoic blocks may have been pulled away from the original craton to form inliers within the orogeny. It is important to note that dispersal of decratonized blocks from the SFC was more effective along the southern and central zones of the Borborema Province, where evidence for Cariris Velhos extensional events has been better defined.

Decratonization related to Neoproterozoic extension and juvenile magmatism could have been widespread throughout West Gondwana, related to the break-up of Rodinia, although the position of the SFC in this supercontinent is contentious^63^. For example, it might account for thinning of the cratonic lithosphere in the Ribeira and Araçuaí belts of southeastern Brazil^64^, decratonizing the eastern margin of the SFC, preparing it for later involvement in the transcurrent Brasiliano tectonics^65^. In the Araçuaí Belt fissural mafic magmatism, associated with the Bahia-Gangila LIP event, preceded the opening of the Adamastor Ocean starting at *ca.* 0.9 Ga^45,66^ with subsequent *ca.* 0.87 Ga rift-related, A-type continental plutonism^31^. Further south, in the Dom Feliciano-Kaoko belt, rift-related siliciclastic and bimodal volcanic rocks preserved in the Neoproterozoic schist belts, from both the Rio de la Plata/Paranapanema and Congo/Kalahari cratonic margins, suggest continental rifting between 0.9 and 0.78 Ga^67^. In the African side of these orogens (*e.g.,* Gariep, Kaoko and Damara-Lufilian belts) extensional tectonics and breakup of surrounding cratons are also constrained to between 0.85 to 0.77 Ga^68–70^. These protracted extensional events preceding the Brasiliano/Pan-African orogens of coastal South America and African equivalents, disrupted and weakened the surrounding cratons, enabling orogenic reworking and transcurrent dispersal of old terranes such as the São Luiz, Curitiba, and Cabo Frio terranes^71,72^. The same could account for Archean/Paleoproterozoic lithosphere in the West Gondwana Orogen in Africa where 1.1–1.0 Ga extension in the Tuareg Shield allowed reworking during the Pan-African Orogeny^2^.

Global estimates for the construction of Gondwana between 0.6 and 0.5 Ga indicate only minor mantle addition^73^, in accordance with our observations in the Borborema Province. The final stage of reworking and transpression was accompanied by voluminous syn-transcurrent high-K calc-alkaline magmatism ranging from mafic to felsic rocks from 0.59 to 0.56 Ga^74–76^, peaking at *ca.* 0.58 Ga (Fig. 2B). Their strongly negative ε_Nd_ values (Fig. 2B) and radiogenic Sr isotopes suggest a metasomatized, enriched lithospheric mantle source^74^. Lead isotopic ratios provide complementary information, suggesting involvement of asthenospheric fluids, possibly responsible for triggering voluminous lithospheric melting^76^. The orogenic collisional period of the BP resulted in crustal thickening, especially along the West Gondwana Orogen^27^, however, Rayleigh wave tomography indicates that this orogen is marked by thinner lithosphere today^77^, thus suggesting that the orogenic roots have been removed after collision. Since this magmatism from 0.59 to 0.56 Ga is contemporaneous with transcurrent deformation, post-dating the collisional event recorded by the West Gondwana Orogen, but synchronous to the collision along SBO, their origin can be attributed to the disturbance of the lithospheric mantle, including asthenospheric fluid influx^76^, unrelated to subduction processes^78^. This disturbance alongside collisional belts might be caused by lithospheric delamination, due to eclogitization at the base of the crust or by convective removal of a thickened thermal boundary layer^79^. The crustal structure of the province revealed by receiver functions and surface wave dispersion suggest that the delamination of the thickened lower crust might have occurred after the collisional period^80^ in combination with asthenospheric upwelling^81^. Such delamination, caused renewed lithospheric weakening that facilitated lateral crustal flow^82^ contributing to the processes of escape tectonics and terrane dispersion in the Borborema Province^19^ (cross-section (c) to (d) in Fig. 6B). Finally, Ar–Ar cooling ages and U–Pb zircon emplacement ages (Fig. 2B) of poorly deformed to isotropic granitoids indicate slow cooling rates with continuous heat supply related to the delamination process until the Cambrian (0.52–0.50 Ga)^19^.

We conclude that the cratonic roots of the São Francisco Craton/Benino-Nigerian Shield, responsible for craton integrity, were weakened by Tonian-age extension. This created the conditions required for the involvement and dispersal of decratonized inliers within the Brasiliano orogen. We suggest that this may have been the general sequence of events for many of the Brasiliano/Pan-African orogens, where extension related to the break-up of cratonic masses and opening of oceanic realms with varying degrees of maturity were followed by convergence, wrench tectonics and late post-collisional magmatism during Gondwana amalgamation.

## Methods

### Nd isotopic maps

In order to discriminate terranes with similar signatures, a large compilation comprising 360 zircon U–Pb ages and 1331 Sm–Nd whole-rock isotope analyses of samples from the BP and the northern SFC were used (Fig. 1B). Sm–Nd isotope distribution of a large number of samples is suitable to identify correlated terranes due to resistance of the Sm–Nd isotope system to post-crystallization thermal disturbance^83^. Data were downloaded from the open sources DateView^84^ and the Geological Survey of Brazil database (http://geosgb.cprm.gov.br). Due to the scarcity of U–Pb crystallization ages from the Benino-Nigerian Shield, for this region we used individual zircon ages for dated metaigneous rocks available in the global zircon compilation in^85^. The compilation was augmented with data from the literature. Most of the data are from (meta)igneous rocks with subordinated input from metasedimentary rocks.

In general, Nd model ages (T_DM_) do not correspond to a specific crust-formation event but instead reflect mixing of material derived from the mantle at different times and are determined by calculating the time when a sample had an isotopic composition identical to that of its source^86^, so they can be understood as minimum ages of crust formation. For Fig. 1B we used Sm–Nd T_DM_ ages as originally reported by different authors (Supplementary Data 1). The Sm–Nd T_DM_ ages were gridded in the ArcGIS software using Inverse-Distance-Weighted Interpolation (IDW). Since significant discontinuities modify the surrounding geological environment, we define the major shear zones as interpolation barriers. We also applied the Gaussian low-pass filter to attenuate high frequencies due to variable spacing between samples. We compared compiled U–Pb zircon crystallization ages and freely available geological maps (http://geosgb.cprm.gov.br) of the Northern SFC and BP to cross-check terrane affinity based on the Sm–Nd T_DM_ map, to identify old terranes within the Borborema orogenic province. From the compiled Sm–Nd dataset, only data with reported ppm contents of Nd, Sm and ^143^Nd/^144^Nd ratio associated with reliable reported U–Pb ages for the magmatic crystallization, were considered for building the ε_Nd(t)_ maps and further crustal growth curve for the Borborema Province. This resulted in 889 data points with assigned geographical position and recalculated ^147^Sm/^144^Nd ratio. A new screening was applied to eliminate unreliable ^147^Sm/^144^Nd resulting in the 837 data points that were used for further gridding of ε_Nd(t)_ values, as described above (Fig. 3). The gridded values resulted in a map with 46,426 pixels with a pixel size of 3.2 × 3.2 km (pixel area = 10.24 km^2^). These pixels where clipped to the igneous crystalline area of the Borborema Province, excluding the Neoproterozoic metasedimentary belts, Phanerozoic basins and more recent cover, resulting in a map with 17,235 pixels with a total area of 176,486 km^2^. Pixel values were further binned to 100 Myr intervals, from 0.5 to 3.4 Ga, along with the U–Pb crystallization ages from individual rock samples. The ε_Nd(t)_ cut-off value of zero was used to distinguish juvenile addition of new crust from reworked crust in the Borborema Province through time. The pixels with ε_Nd_ > 0 were binned to 100 Myr and the cumulative percentage area covered by juvenile rocks was used to infer the crustal growth of Borborema Province from 0.5 to 3.4 Ga. Supplementary Figure S1 shows the data distribution used to grid the Sm–Nd T_DM_ ages.

### Magnetic maps

The airborne magnetic database comprises data from seven surveys between 2001 and 2010, with 500 m flight-line spacing in the N–S direction and flight height of 100 m (http://geosgb.cprm.gov.br). The Total Magnetic Intensity map (TMI) was created by interpolating the magnetic data into a 125 m grid cell size using the bi-directional method and subsequently filtered by a Gaussian low-pass filter. To highlight the regional tectonic framework, we calculated the first vertical derivative of TMI (1VD).

## Supplementary Information

Supplementary Information

Supplementary Data 1

Supplementary Data 2

## Data Availability

All isotopic data used during the study are available in Supplementary Information file (Supplementary Data 1 and 2). Airborne geophysical data from the Geological Survey of Brazil used in Figs. 1B and 4A, and geological maps used in Fig. 1A are freely available at http://geosgb.cprm.gov.br. P-wave tomography model for the Borborema Province in Fig. 1C is available in reference^35^.
