# Supplementary material for: Decratonization by rifting enables orogenic reworking and transcurrent dispersal of old terranes in NE Brazil
Source: Sci Rep. 2021 Mar 11;11:5719. doi: 10.1038/s41598-021-84703-x (PMC7970994; doi:10.1038/s41598-021-84703-x)
Supplement: Supplementary file 1 — Supplementary Information [file 41598_2021_84703_MOESM1_ESM.docx]

**Supplementary material**

**Decratonization by rifting enables orogenic reworking and transcurrent dispersal of old terranes in NE Brazil**

Carlos E. Ganade*^1^, Roberto F. Weinberg^2^, Fabricio A. Caxito^3^, Leonardo B. L. Lopes^1^, Lucas R. Tesser^4^, Iago Costa^1^

^1^Geological Survey of Brazil, Rio de Janeiro, Brazil.

^2^School of Earth, Atmosphere and Environment, Monash University, Clayton, Australia.

^3^Universidade Federal de Minas Gerais, Belo Horizonte, Brazil.

^4^Universidade de São Paulo, São Paulo, Brazil.

*Correspondence to carlos.ganade@cprm.gov.br

**
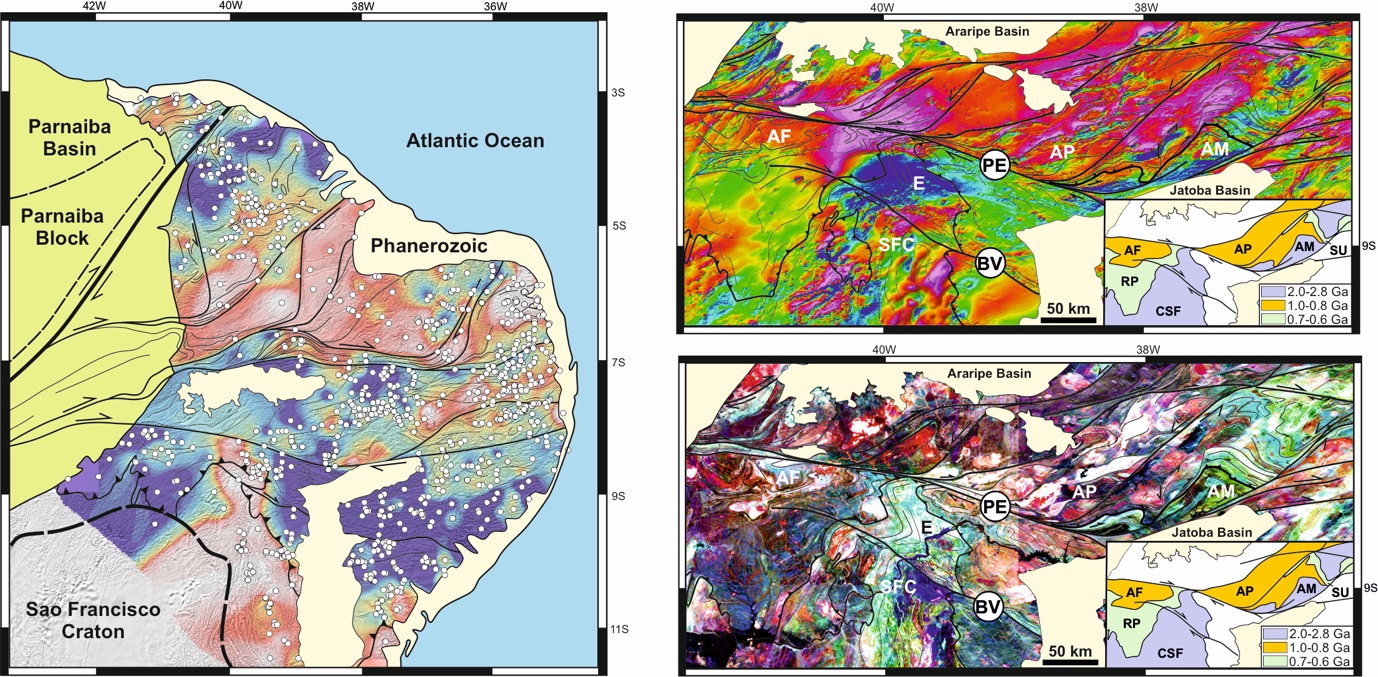
**

**Figure S1 – A.** Sm-Nd T_DM_ age isotopic map of the Borborema Province and northern edge of the São Francisco Craton with magnetic first derivative image as background. White dots represent the location of the compiled samples used in the Inverse-Distance-Weighted Interpolation (IDW). **B.** Total magnetic count image of the investigated area. Note the similar signatures (blueish tones) for the Entremontes (E) and Alto Moxotó (AM) terranes**. C.** Gamma-spectrometric ternary (K, U, Th) image of the investigated area. Note the similar high-Th signatures (greenish tones) of the São Francisco craton (SFC), Entremontes block (E), and Alto Moxotó terrane (AM).

**Supplementary Data 1 –** Compiled zircon U-Pb and whole-rock Sm-Nd data for the Borborema Province.

**Supplementary Data 2 –** Compiled detrital zircon U-Pb ages for central and southern Borborema Province.
